# Cross-Modal Transfer of the Tilt Aftereffect From Vision to Touch

**DOI:** 10.1177/2041669516668888

**Published:** 2016-10-03

**Authors:** Dafni Krystallidou, Peter Thompson

**Affiliations:** Department of Psychology, University of York, UK

**Keywords:** adaptation or constancy, frames of reference, haptics or touch, visuohaptic interactions

## Abstract

Visual input powerfully modulates the dynamics of tactile orientation perception. This study investigated the transfer of the tilt aftereffect (TAE) from vision to somatosensation. In a visual tilt adaptation paradigm, participants were exposed to clockwise or anticlockwise visual tilt, followed by three brief tactile two-point stimuli delivered on their forehead. In a two-alternative forced choice task, participants had to indicate whether the haptic stimulus was tilted to the right or left. Repeated exposure to oriented visual gratings produced a tactile TAE, such that the subsequent tactile stimuli appeared tilted toward the opposite direction. To assess the origin of this effect, the experiment was repeated with the head tilted. Adaptation to a gravitationally tilted grating but with the head tilted so that the grating was retinally vertical induced a robust tactile aftereffect suggesting that the visuotactile TAE is due to spatiotopic rather than retinotopic adaptation.

## Introduction

Visual aftereffects refer to adaptation-induced illusory changes in visual perception and provide a powerful tool for elucidating the neural locus of complex visual processing (Zhao, Seriès, Hancock, & Bednar, 2011). For example, in the case of the tilt aftereffect (TAE), prolonged exposure to tilted visual gratings (10°–30° from vertical) causes a subsequently presented vertical line or grating (test stimulus) to be perceived as being tilted in the opposite direction (Gibson & Radner, 1937). This perceptual phenomenon has been often attributed to lateral interactions between orientation-selective mechanisms at an early stage of visual processing (Tolhurst & Thompson, 1975). In particular, it has been proposed that prolonged adaptation to oriented visual stimuli induces a repulsive shift in the preferred orientation of orientation-selective cells in V1, causing substantial distortions in participant’s subsequent visual orientation perception (Dragoi, Sharma, & Sur, 2000).

Recent research has suggested that visual adaptation can produce aftereffects in other modalities (see Konkle & Moore, 2009). Konkle, Wang, Hayward, and Moore (2009) demonstrated that visual motion adaptation can produce a motion aftereffect in the tactile domain. Using a visual motion adaptation paradigm, participants were exposed to upward or downward visual motion followed by a brief tactile motion stimulus delivered on their right index figure. In a two-alternative forced choice task, participants had to indicate whether the *tactile sweep* of the stimulus was moving upwards or downwards. Crucially, repeated exposure to visual motion in a given direction (upward or downward) produced a tactile motion aftereffect, such that the tactile motion was perceived in the opposite direction. Similarly, Matsumiya (2013) demonstrated that adaptation to a visually presented face belonging to a specific facial expression (happy or sad) elicits a repulsive bias in the subsequent perception of a haptically perceived neutral face and vice versa. Together, these studies illustrate that tactile processing depends on mechanisms adapted by vision, suggesting that visual stimulation can alter tactile processing.

The existing cross-modal research suggests that visual input powerfully modulates the dynamics of tactile orientation perception (e.g., Beauchamp, 2005). The aim of the present experiments was twofold: first, to determine whether the TAE transfers from vision to the tactile modality, analogously with Konkle et al.’s (2009) motion transfer, and second, to determine whether any cross-modal TAE reflects an early stage, retinotopic visuotactile interaction or a later stage, spatiotopic interaction, anchored in a gravitational (i.e., world centered) frame of reference.

## Experiment 1

### Participants and Method

Experiment 1 had three conditions: baseline, adapt left, and adapt right. The order of conditions was randomized for each of the 13 naïve participants. In all conditions, the two-point orientation discrimination of tactile vertical stimuli was determined (Tong, Mao, & Goeldrich, 2013). In the adapt right and adapt left conditions, the test measurements taken were preceded by an adaptation stimulus, presented for 90 seconds, followed by 15 seconds of tactile tests. Top-up periods of 20 seconds adaptation then alternated with further tactile tests until all the observations had been collected. In the baseline condition, the adaptation stimulus was a small fixation cross on a uniform gray background while in the adapt right and left conditions, the stimulus was a high contrast circular grating, 10° in diameter, 4 cycle/degree, square wave grating oriented 15° to the right or left of vertical. All stimuli were displayed on a standard liquid crystal display screen with 1024 × 768 resolution and 60 Hz frame rate, viewed at 57 cm.

**Figure 1.**
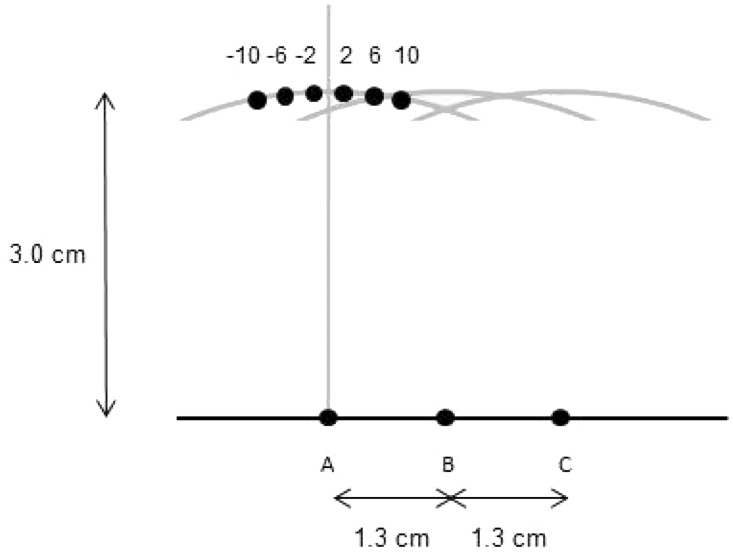
Schematic view of the template (details in the text).

To present the tactile stimuli, we used a plastic template which was used to mark target contact points on the participants’ foreheads. The template consisted of two rows of equidistant holes. The bottom row was composed of three holes placed 1.3 cm apart, which served as the center reference marks. Six equally spaced holes were placed every 4° along the circumference of a 3-cm radius circle centered at each of the three reference points, marking orientations from −10° to 10° in 4° steps (see Figure 1).

Participants were stimulated concurrently at one bottom and one upper point and asked to indicate whether an imaginary line joining the two points was tilted clockwise or anticlockwise from vertical. Each two-point stimulus pair (i.e., combination of upper and bottom points) was presented 5 times for 1 second. Therefore, a total of 15 observations was made at each of six orientations. The order of presentations was randomized. The three bottom points were used to reduce having too many stimulations at a single point on the forehead. (It should be noted that a participant will perceive a stimulus as tilting clockwise when it appears anticlockwise to the experimenter. We reported all responses to be from the participant’s point of view.) A standard two-alternative forced choice psychophysical method with constant stimuli was used. Psychometric functions were plotted for each participant, and the point of subjective vertical (PSV) determined by fitting a cumulative normal distribution to the data; 95% confidence intervals were fitted by a standard bootstrapping procedure.

### Results

**Figure 2.**
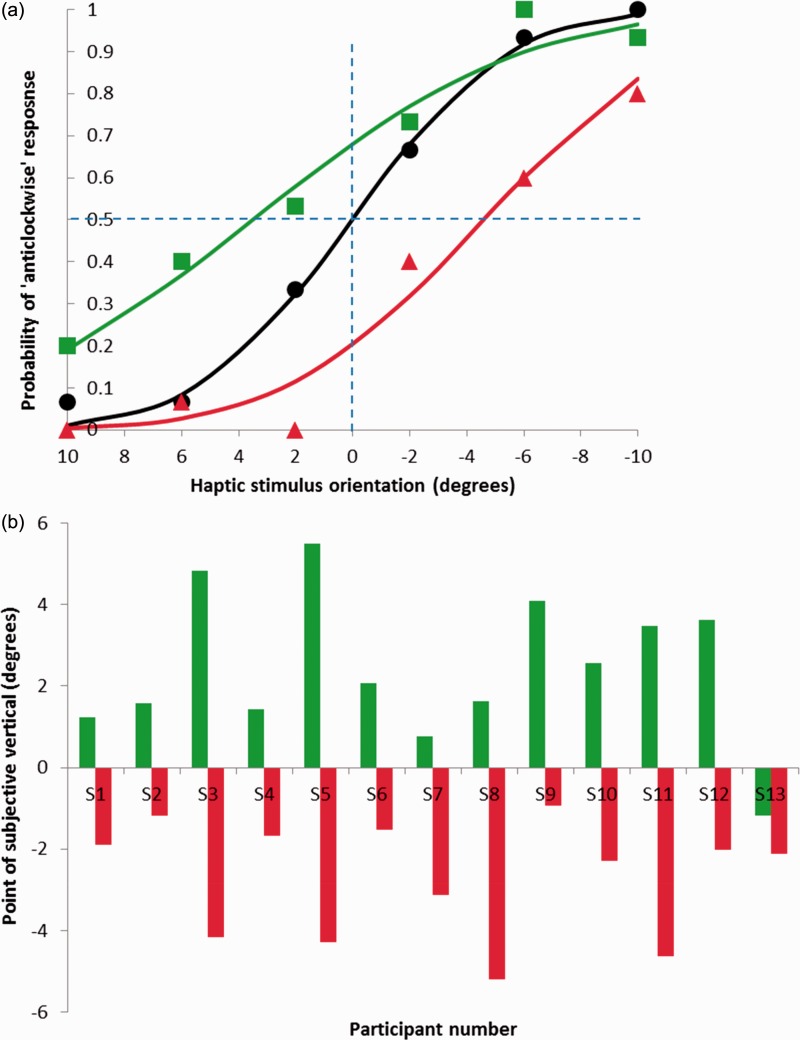
(a) Results for one representative participant. Black circles: baseline judgment of tactile orientation. Green squares: judgments after clockwise adaptation. Red triangles: judgments after anticlockwise adaptation. Note that the *x*-axis plots the perceived orientation from the participant’s viewpoint. Positive numbers refer to clockwise tilt from participant’s viewpoint. Other details in text. (b) Points of subjective vertical for all participants following adaptation to clockwise (green) and anticlockwise (red) gratings. Positive values represent a shift in perceived vertical toward clockwise to the subject but anticlockwise to the experimenter.

Figure 2(a) shows the shifts in perceived vertical before and after adaptation in one participant. After adaptation to a visually presented clockwise-tilted grating (green squares), the participant is more likely to feel a truly vertical stimulus as tilted anticlockwise. The PSV has shifted to +3.5°. Adaptation to a visually presented anticlockwise-tilted grating produces the opposite effect (red triangles), the PSV now being −4.6°. The baseline condition (black circles) shows that this participant was largely unbiased, the PSV being at −0.02°. Results for all 13 participants are shown in Figure 2(b).

These results demonstrate that adaptation to a tilted visual stimulus can shift our perception of the vertical in the haptic domain just as it can in the visual domain—the traditional tilt aftereffect. For all 13 participants, visual adaptation to anticlockwise-tilted gratings caused the subsequent tactile test targets to be perceived as being tilted clockwise to the participant. This leads to the PSV being shifted to a truly anticlockwise tilt. Of the 13 participants, 12 participants perceived this cross-modal aftereffect after adaptation to clockwise visual tilt. On average, the haptic PSV was shifted 2.7° anticlockwise of vertical (from the participant’s viewpoint) after anticlockwise visual tilt adaptation and 2.4° clockwise of vertical after clockwise visual-tilt adaptation.

To compare statistically the effect of adapting stimulus orientation on haptic perception of verticality, a one-way repeated-measures analysis of variance was performed in which the factor of interest was the visual adaptation condition (right-visual tilt, left-visual tilt, no adaptation). Mauchly’s test of sphericity indicated that the assumption of sphericity had been violated (*x*^2^(2) = .50, *p* = .021), and therefore, degrees of freedom were corrected using the Greenhouse-Geisser estimates of sphericity (ɛ = .67). There was a significant main effect of adaptation condition on subsequent haptic verticality perception, *F*(1.33, 15.95) = 41.91, *p* < .001. Planned contrasts revealed that the postadaptation PSV for both *right-tilted*, *F*(1, 12) = 24.97, *p* < .001, and *left-tilted* adapting visual gratings, *F*(1, 12) = 41.65, *p* < .001, was significantly different from the unadapted baseline perceptual boundary. A paired-samples *t* test confirmed that the TAE was not significantly different in magnitude after adaptation to right visual tilt than after adaptation to left visual tilt (Mean = 2.43, *SEM* = 0.51; Mean = 2.69, *SEM* = 0.39), *t*(12) = −0.48, *p* = .638.

## Experiment 2

To further investigate the origin of this cross-modal TAE, we asked whether it reflects a low-level integration of visuotactile orientation signals or a higher order perceptual phenomenon. We tested this hypothesis by examining the reference frame of the visuotactile TAE. It is generally assumed that the visual TAE occurs at low-level visual stages and is thus tied to a purely retinotopic frame of reference (Mathot & Theeuwes, 2013). However, recent experiments on trans-saccadic integration may have demonstrated that orientation shows spatiotopic adaptation, implying that under certain circumstances the TAE operates in gravicentric (allocentric) coordinate frames (Melcher, 2005; Zimmermann, Burr, & Morrone, 2011), though this claim is not uncontroversial, for example, Knapen, Rolfs, Wexler, and Cavanagh (2010). On the other hand, haptic orientation perception, it is generally agreed, is largely governed by egocentric frames of reference (Coleman & Durgin, 2014). Recent research by Mikellidou, Cicchini, Thompson, and Burr (2015) suggests that the *oblique effect*—reduced orientation discrimination for oblique orientations—has both egocentric and allocentric components (Mikellidou et al., 2015).

To determine the reference frame of the visual-to-tactile TAE, we introduced a discrepancy between the gravitational and the egocentric visual vertical by having observers adapt to visual gratings with their heads tilted, by the same angle and direction as the visual adaptor, so that the visually projected gratings were vertical on the retina but gravitationally they remained tilted. If such visual adaptation produces a tactile tilt aftereffect then it would be evidence that the cross-modal effect found in Experiment 1, at least in part, reflects adaptation in a gravitationally based frame of reference. If however a TAE is not observed in this experiment, then this would suggest that the cross-modal TAE documented in Experiment 1 represents a low-level visuotactile integration operating in retinotopic head-centered coordinates.

### Participants and Method

Fourteen participants, of whom 13 had taken part in Experiment 1, completed the experiment. The procedure was identical to Experiment 1 with the single difference that when adapting to the 15° clockwise- (or anticlockwise-) tilted stimuli, the participant’s head was tilted at 15° in the same direction so that the adaptation grating was vertically oriented in retinal coordinates. To control the head tilt of the participants, a 15° tilted headrest was used which constrained the head position to 15° from vertical. (After the experiment, we photographed head positions with the headrest and found deviations from 15° of no more than +/−2°). The postadaptation measures of the haptic vertical were carried out with the head vertical, as in Experiment 1. For those participants who had taken part in Experiment 1, the baseline measures were reused, for the one new participant, baseline measures were taken. In a between-subjects design, eight participants (participant numbers 1–8) adapted to clockwise tilt, while the remaining six participants (participant numbers 9–14) adapted to anticlockwise tilt.

### Results

**Figure 3.**
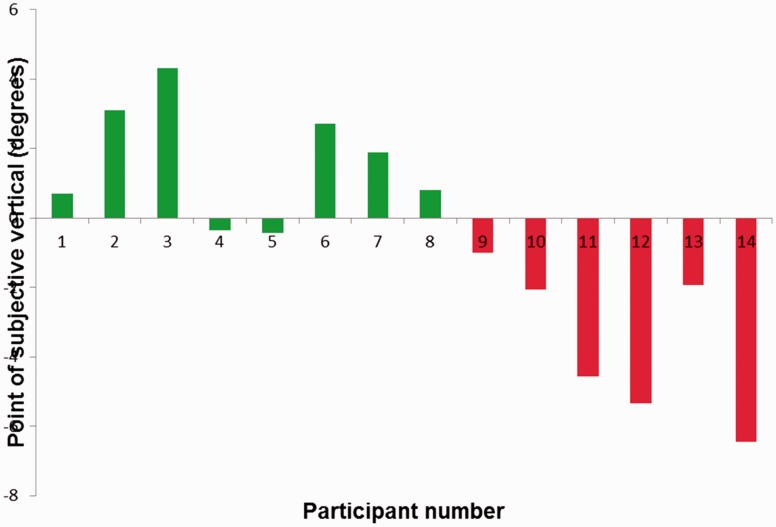
Haptic tilt aftereffects following adaptation to retinally vertical but gravitationally tilted visual gratings. Participants (1–8) adapted to clockwise tilt (green bars), and participants (9–14) adapted to anticlockwise tilt (red bars).

As in Experiment 1, psychometric functions were fitted to the individual data and the PSVs determined (see Figure 3). Six of the eight participants who were adapted to a real-world clockwise tilt experienced a tilt aftereffect (on average 1.6°) qualitatively similar that experienced in Experiment 1. All of the participants who were adapted to an anticlockwise tilt experienced a tilt aftereffect, average size 3.6°. The average size of the effect overall of 2.6° is similar to the effects found in Experiment 1.

A two-tailed paired samples *t* test indicated that adapting to tilted visual gratings while having the head tilted at the same angle significantly shifted participants’ haptic PSV (*p* = .012). To further assess the reliability of this aftereffect, the magnitude of the PSV shift was compared with the visuotactile TAE documented in Experiment 1. For each of the participants who participated in both experiments, the size of the TAE in Experiment was compared with the equivalent magnitude in Experiment 1. A two-tailed paired *t* test indicated no difference between the magnitude of the aftereffect in the two experiments (*p* = .982). As 13 of our participants provided data for both Experiments 1 and 2, we were able to run a correlation between their individual results in the two experiments. The correlation of 0.85 is highly significant (*p* < .01), confirming that the effect size in Experiments 1 and 2 is similar.

## Experiment 3

One intriguing experimental prediction can be made from the result of Experiment 2. It is well known that adapting to a gravitationally vertical stimulus with one’s head tilted leads to a visual TAE (Rieser & Banks, 1981). However, if it is true that the cross-modal aftereffect described here is gravitationally based, as suggested by Experiment 2, then this adaptation, while producing a visual aftereffect, should produce no haptic tilt aftereffect.

### Participants and Method

Fifteen participants undertook three conditions, each in a pseudorandomized order. Baseline measures were taken as described in Experiment 1. The adaptation condition was identical to the adaptation in Experiment 2 except that the adaptation stimulus was oriented vertically. That is, the adaptation grating was presented vertically in space but tilted on the retina. Six of the participants adapted with clockwise tilted head, and nine participants adapted with anticlockwise tilted head. The third condition repeated the adaptation condition but with no adaptation stimulus shown on the screen. Participants who had adapted with clockwise head tilt in the experimental condition adapted with clockwise head tilt in the control condition. This controlled for any effect of head tilt alone.

### Results

**Figure 4.**
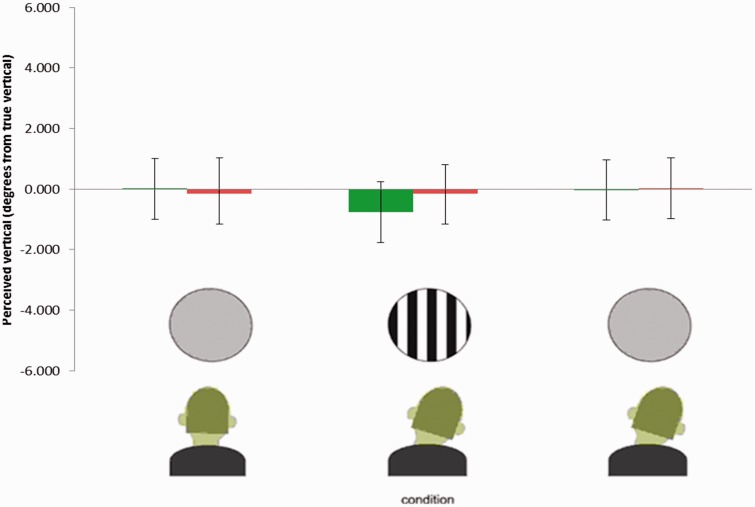
Results of Experiment 3. Error bars show 95% confidence intervals. As earlier, participants adapted with clockwise head tilt shown in green bars, anticlockwise in red bars.

As in Experiment 1, psychometric functions were fitted to the individual data and the PSVs determined. The average baseline setting for all 15 participants was −0.01°, while the average baseline after adapting to a blank screen with head tilt was 0.01°. The data plotted in Figure 4 show the average baseline and postadaptation values of perceived true vertical. A *t* test shows that the difference between the adaptation conditions, adapting head tilt left and head tilt right, are not significantly different (*t*(8) = −0.55, *p* = 0.59).

## Discussion

The present results demonstrate that tactile orientation processing is susceptible to adaptation in the visual modality. In Experiment 1, adaptation to visual tilt of a particular orientation (right or left) induced a robust tactile tilt aftereffect, biasing the haptic PSV away from the orientation of the visual adaptor. Experiment 2 established that the visual-to-tactile TAE is defined in a gravitational frame of reference; adaptation to tilted visual gratings with the head tilted by the same angle produced a powerful haptic TAE. The cross-modal aftereffect documented in Experiments 1 appears to be of similar magnitude to the standard TAE (Gibson & Radner, 1937). Experiment 3 provided confirmatory evidence that the cross-modal aftereffect reflects gravitational orientation rather than retinal orientation.

The results from this study are consistent with the reviewed literature on the cross-modal transfer of aftereffects between vision and somatosensation. Konkle et al.’s (2009) experiment illustrated robust cross-modal motion aftereffects operating both from vision to touch and from touch to vision. Similarly, Matsumiya (2013) reported evidence for a genuine bidirectional visuotactile face aftereffect. Importantly, this study extended recent findings of visuotactile aftereffects in the perception of haptic orientation. The current experiment revealed that haptic orientation perception is dynamically altered by prior exposure to visual tilt, whether visual tilt can similarly be altered by haptic tilt remains to be seen.

The TAE has been extensively investigated in the visual modality and is generally assumed to originate from lateral inhibitory interactions between orientation-selective neurons of the primary visual cortex (e.g., Tolhurst & Thompson, 1975). Nevertheless, the occurrence of a visually induced haptic TAE demonstrates that the neural processing of tactile orientation depends on neural substrates adapted by vision. Further, given that the reference frame of this TAE is gravicentric, it appears that this haptic aftereffect is the result of high-level visual adaptation. The current psychophysical data cannot reveal the locus of this interaction. However, one prominent candidate is the left parieto-occipital complex. This region is thought to be specialized for multisensory spatial processing of orientation (Kitada et al., 2006). Indeed, Sathian, Zangaladze, Hoffman, and Grafton (1997) found that this region was activated during tactile discrimination of grating orientation. Similarly, TMS research by Zangaladze, Epstein, Grafton, and Sathian (1999) indicated that stimulation over this locus interferes with haptic discrimination of grating orientation. The same region has been shown to be activated during visual grating orientation discrimination (Sergent, Ohta, & MacDonald, 1992). More recently, Kitada et al. (2006) confirmed these findings in a functional magnetic resonance imaging study. Ultimately, evidence for tactually evoked activation of visual parieto-occipital cortical region provides strong evidence for the existence of partially overlapping, shared neural substrates for visuotactile orientation perception. Nevertheless, the spatial resolution of current functional magnetic resonance imaging technology is coarse, and a typical voxel might include a few million neurons (Logothetis, 2008). As a result, it is possible that seemingly identical regions activated in visual and haptic orientation tasks are in fact distinct neural populations.

In conclusion, the present findings suggest that vision strongly alters haptic orientation processing. Using a tilt adaptation paradigm, we showed that adaptation to visual tilt results in a robust tactile TAE—the perception of a subsequent oriented tactile target in the opposite direction to the visual adaptor. Further, we showed that this visually induced tactile aftereffect operates in allocentric frames of reference. It is worth noting that the tactile aftereffect on the forehead has a polarity as seen from *inside* the head—that is, adaptation to a visual pattern tilted clockwise makes a vertical tactile stimulus appear tilted clockwise from the experimenter’s viewpoint but anticlockwise from the participant’s viewpoint. This experience concurs with a well-known demonstration: asked to write a word on your forehead you write it in mirror writing, that is, it is written as if seen from inside the head. Writing a word on the back of your head is not so transformed. Our experiments may have been easier if we had probed the tactile effects of the visual adaptation on the back of the neck!

The present findings suggest that the haptic orientation processing system is influenced by high-level visual representations. Nevertheless, the current psychophysical results cannot reveal the exact locus underlying this visuotactile interaction. Perhaps the investigation of the cross-modal TAE using advanced functional-imaging methods will aid our understanding of this intriguing aftereffect, leading to further advances in the complex dynamics of multisensory orientation processing.
